# Association of Christian Orthodox Fasting with Sociodemographic, Anthropometric and Lifestyle Factors and Serum Biochemical Indices: A Cross-Sectional Study on Patients with Metabolic Diseases

**DOI:** 10.3390/metabo14010067

**Published:** 2024-01-19

**Authors:** Nikolaos E. Rodopaios, Efthymios Poulios, Sousana K. Papadopoulou, Olga Alexatou, Alexandra-Aikaterini Koulouri, Anthony G. Kafatos, Vasileios Papaliagkas, Evmorfia Psara, Anastasia Giannakoula, Gerasimos Tsourouflis, Georgios Antasouras, Constantinos Giaginis

**Affiliations:** 1 Department of Social Medicine, Preventive Medicine and Nutrition, Medical School, University of Crete, 70013 Heraklion, Crete, Greece; nrodopaio@past.auth.gr (N.E.R.); alexkoulou@yahoo.com (A.-A.K.); kafatos@med.uoc.gr (A.G.K.); 2 Department of Food Science and Nutrition, School of the Environment, University of the Aegean, 81400 Myrina, Lemnos, Greece; epoulios@aegean.gr (E.P.); rd.olga.alexatou@gmail.com (O.A.); fnsd21013@fns.aegean.gr (E.P.); g.antasouras@gmail.com (G.A.); 3 Department of Nutritional Sciences and Dietetics, School of Health Sciences, International Hellenic University, 57400 Thessaloniki, Greece; souzpapa@gmail.com; 4 Department of Biomedical Sciences, School of Health Sciences, International Hellenic University, 57400 Thessaloniki, Greece; vpapaliagkas@gmail.com; 5 Department of Agriculture, International Hellenic University, Sindos, 54700 Thessaloniki, Greece; agianna@ihu.gr; 6 Second Department of Propaedeutic Surgery, Medical School, National and Kapodistrian University of Athens, 11527 Athens, Greece; gtsourouflis@med.uoa.gr

**Keywords:** Christian Orthodox fasting, metabolic disorders, Mediterranean diet, religious fasting, metabolic health, cardiometabolic diseases, obesity, dyslipidemia, blood pressure, serum glucose

## Abstract

Christian Orthodox fasting, a type of time-restricted diet, which presents some similarities to the Mediterranean Diet, also including certain similarities with periodic vegetarianism or other time-restricted diets (e.g., intermittent diet and Ramadan fasting), may cumulatively be related to the same or even better beneficial healthy effects as these well-recognized dietary patterns. The present study aimed to explore the potential beneficial impact of Christian Orthodox fasting in patients with metabolic disorders, such as diabetes mellitus type 2, excessive obesity, hypothyroidism and osteoporosis. This was a cross-sectional study, including 135 patients with metabolic disorders (67 fasters and 68 non-fasters). The enrolled fasters had adapted Christian Orthodox fasting recommendations for at least twelve consecutive years or even from childhood. Relevant questionnaires were used to record sociodemographic, anthropometric and lifestyle data of the study population through face-to-face interviews between the enrolled individuals and qualified personnel during a non-fasting period. Christian Orthodox fasting patients showed a significantly and independently lower prevalence of overweight/obesity and abdominal obesity, which is highly associated with cardiometabolic disease risks, as well as a significantly and independently lower incidence of hypertension, including separately lower systolic and diastolic pressure, than non-fasting patients. Fasters also had a significantly and independently increased prevalence of an advanced educational level and no smoking history, as well as a lower incidence of sedentary behavior, and a trend of a correlation with reduced c-reactive protein (CRP), an indicator of inflammation, compared to non-fasters. Fasters also exhibited higher serum albumin and high-density lipoprotein (HDL) levels, as well as lower glucose levels, than non-fasters. This is one of the few cross-sectional studies demonstrating that Christian Orthodox fasting may promote metabolic health by improving several aspects of metabolic disorders, being associated with specific sociodemographic, anthropometric and lifestyle factors. Further studies conducted on larger sample sizes from different countries and different ethnicities that include Christian Orthodox fasters are recommended to evaluate the impact of long-term religious fasting effects on human health, either as a preventative factor reducing the risk of chronic diseases and especially cardiometabolic disorders or as a nutritional intervention to ameliorate symptom severity.

## 1. Introduction

The Mediterranean diet (MD) constitutes one of the most well-recognized dietary patterns, which has been increasingly found to exert diverse beneficial effects on human health, promoting a great daily quality of life and healthy aging, as well as increasing life expectancy [1,2]. The MD has been considered a beneficial dietary pattern by decreasing the risks of a variety of human disorders and pathological states, including metabolic disorders [3,4,5]. The MD, initially invented by Ancel Keys [6] in 1960, is a plant-based diet comprising high amounts of vegetables, fruits, legumes, wholegrain cereals, nuts and extra virgin olive oil, the moderate consumption of dairy products, seafoods and poultry, and the low intake of saturated fats and processed red meat products [7].

The Christian Orthodox fasting dietary pattern has been the origin and the main characteristics of the fasting of the Christian Orthodoxy over 2000 years [8]. Christian Orthodox fasting constitutes the interchange of a vegetarian diet with the additional inclusion of fish, seafood and snails (as a source of proteins) during fasting periods, to a mixed diet that also includes meat, dairy products and eggs. Extra virgin olive oil is usually allowed on certain days, even during major fasting periods [9]. Christian Orthodox fasting lasts for a total of 180–200 days annually and takes place during four major periods (Nativity, Lent, Assumption and Saint Apostles fasting), in which different foods are prohibited during each fasting period [9,10]. In addition, it takes place on almost every Wednesday and Friday and three other daily feasts (5 January, 29 August and 14 September) [9,10].

Based on the above, Christian Orthodox fasting is a traditional type of a time-restricted diet, which constitutes a distinct dietary pattern beyond the MD and plant-based diets and which permits the consumption of seafoods and snails. The above foodstuffs prevent potential nutritional deficiencies of essential nutrients, which are not synthesized by the human body, such as the essential fatty acids and vitamin B12 [11]. Moreover, Christian Orthodox fasting does not include ultra-processed foods, which are highly related to an increased risk of diet-related non-communicable diseases [12]. Even though a considerable positive association was recorded between the national household availability of ultra-processed foods and the national prevalence of obesity among adults, ultra-processed products dominate the food supplies of high-income countries, and their consumption is now rapidly increasing in middle-income countries [12,13]

Christian Orthodox fasting, as a distinct, traditional type of time-restricted fasting, has been compared to the MD and periodic vegetarianism due to its certain similarities with these dietary patterns [14,15]. Christian Orthodox fasting practices also adhere to the World Cancer Research Fund’s Cancer Prevention Recommendations because of the high intake of fiber-rich foods, fruits, vegetables and legumes, low intake of refined carbohydrates and low consumption of meat products and dietary saturated fatty acids [9,10]. Notably, religious fasting, particularly Christian Orthodox and Ramadan Islamic fasting, are considered sustainable diets, as their recommendations may have a positive impact on planetary health [16]. Christian Orthodox fasting has also been shown to exert potential preventive effects against obesity, cardiometabolic risk factors and diabetes mellitus type 2 [10,17]. In the last few years, Christian Orthodox fasting has been reported to exert beneficial effects concerning glucose, lipids and body weight control, being characterized by lower caloric intake, especially during the fasting periods [10,18]. Thus, this type of religious dietary pattern may be of general interest concerning public health promotion. However, there is no adequate research so far concerning the impact of Christian Orthodox fasting in patients with metabolic disorders. In view of the above considerations, the present cross-sectional study aimed to explore the potential beneficial impact of Christian Orthodox fasting in patients with metabolic disorders, such as diabetes mellitus type 2, excessive obesity, hypothyroidism and osteoporosis, focusing on several sociodemographic, anthropometric and lifestyle factors, as well as blood biochemical parameters.

## 2. Materials and Methods

### 2.1. Study Population

This was a cross-sectional study that took place in Athens and Thessaloniki in Greece, which included 135 Greek individuals. Volunteers for the study were recruited by a call for participants that was disseminated at public universities, monasteries and churches, as well as public hospitals. Ethical approval of the study was given by the Bioethics Committee of the Alexander Technological Educational Institute (ethical approval code: 31-5/5679, approval date: 17 December 2013). All enrolled individuals had been diagnosed with a metabolic disorder, such as hypothyroidism, osteoporosis, diabetes mellitus type 2 or excessive obesity. All participants’ information was confidential, and all participants were informed about the purpose of the study and signed a written consent form, accepting their data to be published. In our study, we carried out all the guidelines of the Declaration of Helsinki in accordance with the World Health Organization (52nd WMA General Assembly, Edinburgh, Scotland, 2000).

Among the 135 individuals, 67 of them were fasters who had adapted Christian Orthodox fasting recommendations based on the fasting calendar, which spans 180–200 days of the year, from their childhood or at least for the last twelve consecutive years. We implemented this cut-off of twelve years since it was easier to enroll individuals with this fasting duration and this is simultaneously an adequate period of fasting adherence to derive more accurate results. The remaining 68 participants were non-fasters and were age-matched with fasting participants, and they did not follow the same dietary pattern and did not abstain from any food item (e.g., lactose, wheat, etc.) due to medical and/or lifestyle reasons during the last 20 years. Exclusion criteria were (a) participants being ˂18 years or >65 years, (b) not providing written informed consent, (c) not participating in the scheduled appointment to collect measurements, (d) having non-communicable diseases (NCDs), such as cardiovascular diseases, cancer, neurodegenerative diseases and other, (e) having food allergies and (f) being pregnant or breastfeeding.

### 2.2. Sociodemographic, Anthropometric and Lifestyle Factor Collection

Relevant questionnaires were used to record sociodemographic, anthropometric and lifestyle data of the study population through face-to-face interviews with qualified personnel. Participants’ sociodemographic characteristics, such as age, gender, educational level and marital status, were self-reported during face-to-face interviews between the enrolled individuals and qualified personnel (medical and nursing personnel, nutritionists and dietitians) in an isolated and relaxed environment to reduce recall bias. The educational level was categorized into three classes: (a) primary education, (b) secondary education and (c) university studies.

Participants’ anthropometric parameters, such as body weight, waist and hip circumference and height were measured at the time of the study by qualified personnel. Body weight was measured using the same electronic scale, and height was measured using a portable stadiometer. The body weight was measured to approximately the nearest 100 g, and the height was measured to approximately the nearest 0.50 cm. The International Obesity Task Force (IOTF) reference was utilized to define normal weight, overweight and obesity [19,20]. The waist circumference was measured at the midpoint between the lower margin of the last palpable ribs and the top of the iliac crest, being classified as low, medium or high [21]. Waist circumference (WC) is a simple measurement that constitutes one of the criteria for metabolic syndrome and has been well recognized as a useful risk indicator of abdominal obesity, independently of measuring the BMI [22,23]. The Waist-to-Hip ratio (WtHR) was also estimated by dividing the waist measurement by the hip measurement. In this aspect, it should be noted that the WtHR has been considered a superior index to BMI [22,23]. In fact, it has been identified as an effective indicator of abdominal obesity, which is considered a better anthropometric measure for estimating the risk of several cardiometabolic diseases more effectively [24,25]. WtHR was calculated by dividing the WC by the hip circumference, being classified as low, medium or high [24,25].

Lifestyle factors, such as smoking habits, physical activity, sedentary behavior and sleep duration, were self-reported during the face-to-face interviews. Physical activity was classified into four groups, as very low (never or one session of 45 min of weekly physical activity), low (1–2 sessions of 45 min of weekly physical activity), moderate (2–3 sessions of 45 min of weekly physical activity), and high (4 or more sessions of 45 min of weekly physical activity), including both exercise and non-exercise physical activities [26,27,28]. Sedentary behavior was any waking behavior characterized by an energy expenditure ≤ 1.5 metabolic equivalents (METs) in a sitting, reclining or lying posture [28,29,30]. In general, this means that any time a person is sitting or lying down, they are engaging in sedentary behavior, being classified as low, moderate or high [28,29,30]. Common sedentary behaviors include TV viewing, video game playing, computer use (collectively termed “screen time”), driving automobiles and reading [28,29,30]. Sleep duration was classified as normal (7–8 h or abnormal (˂7 h or ˃8 h) [31,32]. Blood pressure was measured with the Omron BP monitor (Omron, Hoffman Estates, IL, USA), and the researcher recorded both systolic and diastolic blood pressure values.

Clarifications of detailed instructions were systematically given to the participants by the qualified personnel regarding the completion of questionnaires, while a detailed presentation of the questions to facilitate accurate answers was performed to increase the validity of participants’ responses. Sociodemographic, anthropometric and lifestyle factors were collected during a non-fasting period.

### 2.3. Laboratory Parameters

Blood biochemical parameter levels, including albumin, total cholesterol, low-density lipoprotein (LDL), high-density lipoprotein (HDL), triglycerides, vitamin D 25-OH, folic acid, vitamin B12, c-reactive protein (CRP), glucose, Ca, alkaline phosphatase (ALP), Fe, creatinine, urea and uric acid, were retrieved from the medical records of the enrolled patients with metabolic disease. Blood biochemical parameter levels were measured during a non-fasting period.

### 2.4. Statistical Analysis

We used Student’s *t*-test for continuous variables that followed the normal distribution using a Kolmogorov–Smirnov test. We applied the Chi-square test for categorical variables. The normally distributed quantitative variables are presented as the mean value ± Standard Deviation (SD), with the qualitative variables as absolute or relative frequencies. We performed multiple logistic regression to assess whether Christian Orthodox fasting is independently associated with sociodemographic, anthropometric and lifestyle factors and serum biochemical indices after an adjustment for potential confounding factors. As confounding factors, we included only those parameters that showed a significant association with Christian Orthodox fasting in the univariate analysis. Multiple regression results are presented as Hazard Ratios (HRs) and 95% confidence intervals (CIs). Differences were considered significant at *p* < 0.05 and a 95% Confidence Interval. Statistica 10.0 software, Europe (Informer Technologies, Inc., Hamburg, Germany), was applied for the statistical analysis of the survey data.

## 3. Results

### 3.1. Descriptive Statistics of the Study Population

#### 3.1.1. Sociodemographic Characteristics

All descriptive statistics of the study population are presented in Table 1. In this cross-sectional study, 135 Greek individuals diagnosed with a metabolic disorder were enrolled (67 fasters and 68 non-fasters) with a mean age of 55.8 ± 7.3 years (range: 35–76 years). Most of them (70.4%) were women, and 29.6% were men. Concerning their educational level, 23.7% had received primary education, 40.7% had completed secondary education and 35.6% of them had accomplished university studies. In addition, most of the enrolled individuals (77.8%) were married, and the remaining 22.2% of them were not married.

#### 3.1.2. Anthropometric Characteristics

Concerning the anthropometric characteristics of the study population, 47.4% of the enrolled individuals were overweight and 39.3% of them were obese based on the BMI classification, while only 13.3% of the participants had a normal weight. Regarding abdominal obesity and WC categorization, 34.1% of the assigned individuals had a medium risk of morbidity and 46.7% had a high risk of morbidity, whereas only 19.2% of them presented with a low risk of morbidity. Concerning abdominal obesity based on the WtHR classification, 43.0% of the enrolled individuals had a medium risk of morbidity and 40.0% of them presented with a high risk of morbidity, whereas only 17.0% of them had a low risk of morbidity.

#### 3.1.3. Lifestyle Characteristics

Most of the assigned individuals (69.6%) were non-smokers, and 30.4% were regular smokers. Classifying the study population according to their physical activity, 41.5% of the participants had very low and 27.4% had low physical activity, whereas 21.5% of them and 9.6% of them reported moderate and high levels of physical activity, respectively. In addition, 20.0% of the enrolled individuals presented with high levels of sedentary behavior, 34.8% of them showed moderate levels and 45.2% of them reported high levels of sedentary behavior.

#### 3.1.4. Disease Classification and Laboratory Parameters

Concerning the type of metabolic disorder, 28.9% of the enrolled individuals suffered from hypothyroidism and 23.0% of them showed extremely high obesity (BMI ≥ 40 Kg/m^2^). In addition, 23.0% of the participants suffered from osteoporosis, and 25.1% of them were diagnosed with diabetes mellitus type 2.

In addition, 58.5% of the assigned individuals were hypertensive and 41.5% of them were normotensive. The mean systolic blood pressure of the study population was 134.6 ± 11.4 mmHg, and the mean diastolic blood pressure was 83.2 ± 5.8 mmHg. The mean serum albumin levels of the participants were 4.2 ± 0.3 g/dL. The mean total cholesterol and triglyceride levels were 205.8 ± 98.7 mg/dL and 179.9 ± 98.7 mg/dL, respectively. The mean LDL and HDL levels were 115.5 ± 43.8 mg/dL and 50.7 ± 16.2 mg/dL, respectively. The mean levels of vitamin D 25-OH, folic acid and vitamin B12 were 17.3 ± 7.4 ng/mL, 2.04 ± 1.71 ng/mL and 315.2 ± 206.1 pg/mL, respectively. The mean levels of CRP, glucose, Ca and ALP were 0.36 ± 0.29 mg/dL, 102.1 ± 35.6 mg/dL, 9.6 ± 1.4 mg/dL and 66.2 ± 18.8 U/L, respectively. Moreover, the mean levels of Fe, creatinine, urea and uric acid were 91.1 ± 28.7 μg/dL, 0.9 ± 0.2 mg/dL, 31.9 ± 14.1 mg/dL and 4.8 ± 1.3 mg/dL, respectively.

### 3.2. Association of Christian Orthodox Fasting with Sociodemographic, Anthropometric and Lifestyle Factors and Serum Biochemical Indices during a Non-Fasting Period

#### 3.2.1. Association of Christian Orthodox Fasting with Sociodemographic Characteristics

In cross-tabulation, the mean participant age was not differentiated between fasters and non-fasters (Table 2, *p* ˃ 0.05). Christian Orthodox fasting was marginally more frequently observed in female than in male participants (Table 2, *p* = 0.0464). Fasters had significantly higher levels of education than non-fasters (Table 2, *p* = 0.0001). Marital status was not different between fasters and non-fasters (Table 2, *p* ˃ 0.05).

#### 3.2.2. Association of Christian Orthodox Fasting with Anthropometric Characteristics

Based on the BMI classification, non-fasters were significantly more frequently overweight or obese compared to fasters (Table 2, *p* = 0.0075). Accordingly, non-fasters had significantly more frequent abdominal obesity than fasters; abdominal obesity was determined based on either the WC index or the WtHR index (Table 2, *p* = 0.0017 and *p* ˂ 0.0001, respectively).

#### 3.2.3. Association of Christian Orthodox Fasting with Lifestyle Characteristics

Fasters were significantly more frequently non-smokers than non-fasters (Table 2, *p* = 0.0018). Accordingly, fasters were significantly characterized by significantly higher levels of physical activity than non-fasters (Table 1, *p* = 0.0192). On the other hand, fasters showed significantly lower levels of sedentary behavior compared to non-fasters (Table 1, *p* = 0.0003). Moreover, fasters significantly showed a marginally higher prevalence of a normal sleep duration compared to non-fasters (Table 1, *p* = 0.0437).

#### 3.2.4. Association of Christian Orthodox Fasting with Blood Biochemical Parameters

In cross-tabulation, fasters showed a significantly lower prevalence of hypertension than non-fasters (Table 1, *p* = 0.0004). Accordingly, fasters had significantly lower systolic and diastolic blood pressure than non-fasters (Table 1, *p* = 0.0001 and *p* ˂ 0.0001, respectively). Serum albumin levels were significantly lower in non-fasters than in fasters (Table 1, *p* = 0.0118). Serum total cholesterol, triglyceride and LDL levels were not significantly differentiated between fasters and non-fasters (Table 1, *p* ˃ 0.05). On the other hand, serum HDL levels were significantly higher in fasters than in non-fasters (Table 1, *p* = 0.0068). Serum CRP levels, an indicator of inflammation, were significantly higher in non-fasters than in fasters (Table 1, *p* = 0.0367). Accordingly, glucose levels were significantly higher in non-fasters than in fasters (Table 1, *p* = 0.0252). All other biochemical parameters were not significantly differentiated between fasters and non-fasters (Table 1, *p* ˃ 0.05).

### 3.3. Multivariate Binary Logistic Regression Analysis of Christian Orthodox Fasting Adjusting for Potential Confounding Factors

In the multivariate binary logistic regression analysis, Christian Orthodox fasting was independently associated with the educational level, smoking habits, sedentary behavior, BMI, WC, WtHR, hypertension, systolic and diastolic pressure, albumin, HDL and glucose levels after an adjustment for potential confounding factors (Table 3, *p* ˂ 0.05). The associations of Christian Orthodox fasting in the unadjusted analysis with participants’ gender, physical activity, sleep duration, serum albumin and CRP levels did not remain significant in the multivariate regression analysis (Table 3, *p* ˃ 0.05).

Participants with a higher educational level who had completed university studies showed an 81% higher probability of adopting Christian Orthodox fasting than those with a lower educational level (Table 3, *p* = 0.0024). Non-smokers exhibited an 72% higher probability of adopting Christian Orthodox fasting than regular smokers (Table 3, *p* = 0.0103). Fasters had a 31% lower likelihood of presenting with sedentary behavior than non-fasters (Table 3, *p* = 0.0185).

Concerning anthropometric factors, non-fasters showed an 82% higher risk of overweight/obesity compared to fasters (Table 3, *p* = 0.0293). Based on WC measurements, non-fasters exhibited an 95% higher probability of abdominal obesity than fasters (Table 3, *p* = 0.0128). Based on WtHR measurements, non-fasters also showed a two-fold higher risk of abdominal obesity than fasters (Table 3, *p* = 0.0002).

Non-fasters had an 67% higher risk of hypertension compared to fasters (Table 3, *p* = 0.0088). Accordingly, non-fasters showed 58% and 72% higher probabilities of increased systolic and diastolic pressure, respectively, than non-fasters (Table 3, *p* = 0.0007 and *p* = 0.0005, respectively). Fasters exhibited an 15% higher probability of presenting with increased HDL levels compared to non-fasters (Table 3, *p* = 0.0231). Moreover, non-fasters showed a 14% higher risk of presenting with increased glucose levels than fasters (Table 3, *p* = 0.0402).

## 4. Discussion

This is one of the few cross-sectional studies that explored the potential beneficial effects of Christian Orthodox fasting on human metabolic health, and especially against specific metabolic disorders, taking into consideration multiple modifiable and non-modifiable factors, such as sociodemographic, anthropometric and lifestyle factors, as well as blood biochemical parameters related to the health status of participants. Patients diagnosed with a metabolic disorder that had adopted the traditional Christian Orthodox fasting for a period of at last twelve consecutive years showed a healthier metabolic status compared to non-fasting patients. Patients adhering to Christian Orthodox fasting had a higher educational level and no smoking history, as well as a lower sedentary behavior, after an adjustment for several confounding factors. To the best of our knowledge, the independent positive associations of this type of fasting with the higher educational level and the lower sedentary behavior in fasters compared to non-fasters are reported for the first time. In additional, non-fasting patients showed a higher prevalence of overweight/obesity and abdominal obesity, as well as a higher incidence of hypertension, including separately higher systolic and diastolic pressure, than fasting patients. The findings of the lower prevalence of abdominal obesity in fasters with metabolic disorders is reported for the first time in patients with metabolic disorders. This finding is very crucial since abdominal obesity is highly associated with several chronic diseases. Fasters also exhibited higher serum albumin and HDL levels, as well as lower glucose levels, in the multivariate analysis. In the univariate analysis, even if Christian Orthodox fasting was also significantly associated with the female gender, higher physical activity levels, better sleep duration and lower CRP levels, these associations did not remain significant after an adjustment for several confounding factors. A reasonable question that is raised is that in multivariate regression analysis, sedentary behavior remained significant, whereas physical activity did not remain significant. However, it should be noted that physical inactivity and sedentary behavior are different constructs [29,30]. This is ascribed to the fact that there is a difference between a person who is sedentary and a person who is physically inactive. More to the point, being physically inactive means not doing enough physical activity (in other words, not meeting the physical activity guidelines), whereas being sedentary means sitting or lying down for long periods [29,30].

This distinct traditional type of fasting between Christian Orthodox individuals has been related to some components and habits of the MD [14], also presenting with certain similarities to periodic vegetarianism [15]. Even if the MD has been considered the most well-recognized dietary pattern related to diverse beneficial effects on human health, promoting quality of life, healthy aging and life expectancy by decreasing the risks of a variety of human disorders, the research concerning the distinct Christian Orthodox fasting remains sufficiently scarce, while the derived previous results are quite inconclusive [9,10]. The traditional fasting habits of Christian Orthodoxy also have certain similarities to other time-restricted diets, such as intermittent fasting and Ramadan fasting. However, there are parallelly several distinct differences among them [33,34,35,36].

In accordance with our findings, a recent study supported evidence for the potential benefits of religious fasting on fasting glucose levels [37]. In this aspect, a cross-sectional study exploring the impacts of Christian Orthodox fasting on several cardiometabolic factors in 50 Athonian monks showed that both serum lipid and glucose levels, as well as the homeostasis model assessment of insulin resistance (HOMA-IR), were within the normal ranges [38]. In addition, during Christmas fasting, a significant decrease in glucose levels was reported at the end of the fasting period [15]. A subsequent survey also compared the diets of 43 males from the general population that regularly fasted (20–45 years) and 57 age-matched Athonian monks, revealing better insulin sensitivity, as assessed based on the HOMA-IR, in Monks compared to that in the general population [38]. On the other hand, two studies showed that no changes in fasting glucose levels were detected during a one-year period or during a 40-day fasting period; however, these studies were conducted on healthy individuals for a rather small period of fasting [39,40]. A more recent study conducted on 176 individuals showed that fasters exhibited significantly higher serum LDL levels than non-fasters, but lower serum glycose and triglycerides levels [18,41]. In this aspect, our data are restricted to serum fasting glucose levels, and thus, we cannot confirm the previously reported results concerning insulin sensitivity. Most of the currently available clinical studies support the evidence that religious fasting may improve glucose control; however, additional studies are recommended to draw definitive conclusions, as the currently available studies have some discrepancies, e.g., different age groups, proportions of enrolled men and women, different fasting durations and different types of citizens (monks or members of the general populations), as well as different sample sizes.

Furthermore, the role of Christian Orthodox religious fasting in lipid control in individuals with and without dyslipidemia has recently been explored [37]. This study showed that Christian Orthodox fasting may decrease HDL, LDL and triglyceride levels, especially in people with dyslipidemia [37]. In this aspect, we found lower serum levels of total cholesterol, triglycerides and LDL in fasters than in non-fasters, albeit at a non-significant level. The above may be ascribed to the different sample sizes and the differences in the metabolic disorders of the enrolled study populations. On the other hand, it should be noted that the fasters from our clinical sample showed significantly higher HDL levels than non-fasters. In contrast, at the end of the fasting period, fasters have been shown to exhibit 12.5% lower total cholesterol levels and 15.9% lower LDL cholesterol levels than non-fasters [40]. This study also showed that the LDL/HDL fraction was lower for fasters; however, any significant differences concerning HDL levels were not identified [38]. Similar findings were observed when the pre-fasting and post-fasting values of fasters were compared, with no differences between fasters and non-fasters [18]. Kokkinopoulou et al. also showed that fasters exhibited significantly higher triglycerides [18]. Fasters also had a significantly lower intake of calories [18,42]. Additionally, in a 40-day (fasting and non-fasting days) survey of 36 nuns and monks, a reduction in total cholesterol, a rise in triglycerides and a moderate drop in LDL/HDL during fasting were noted [41].

Another study conducted on 60 healthy overweight Greek adults aimed to compare the effects of Christian Orthodox fasting on blood lipids during an interval of 7 weeks, revealing considerable reductions in both HDL and LDL levels [43]. Overall, total cholesterol and HDL decreases were more prominent during religious fasting periods, compared to time-restricted fasting, despite similar anthropometric indices [43]. In another study, positive effects on total cholesterol and LDL levels were noted; however, the effect on HDL levels remained unclear [40]. A previous study also indicated caloric restriction during fasting, accompanied by a reduction in fat intake and an increase in carbohydrate and fiber intake [44]. Improvements in blood lipid control have been noted throughout the studies, especially on total cholesterol and LDL levels, whereas inconsistent findings on HDL cholesterol was reported [44]. All of these discrepancies in the lipid profile between the previous studies and the current study may be ascribed to the different characteristics of the enrolled individuals, as some studies included healthy individuals, and others were conducted on patients with dyslipidemia or other metabolic disorders. There is also a difference among the studies concerning participants’ age or lifestyles, as some were performed on monks and others on non-clericals, belonging to the general population.

Furthermore, there are some clinical studies investigating whether Christian Orthodox fasting could influence blood pressure. In this respect, blood pressure changes in 38 religious Christian Orthodox fasters and 29 matched controls living in Crete were followed-up for one year [45]. The relevant data were collected prior to and at the end of the three major fasting periods of the Christian Orthodox calendar (Christmas, Easter and Assumption) [46]. During this survey, fasters exhibited greater mean systolic and diastolic blood pressure than the control group, while the non-fasting period was characterized by a strong effect on decreasing the blood pressure levels of fasters [47]. Near the end of fasting periods, the fasters’ incidence of Christmas and Lent “high-normal” blood pressure was greater than that of the controls, while it was also lowered during Assumption and reached the very low levels of controls. Blood lipids were considerably related to systolic and diastolic blood pressure for most measurements [47]. A recent cross-sectional study on 175 individuals aged >50 years also showed lower systolic and diastolic blood pressure [18]. All of the above results are in line with our findings. However, in another study, systolic, but not diastolic, blood pressure was considerably greater, while the Christian Orthodox religious fasting diet did not appear to lead to an observable impact on blood pressure [48]. The above discrepancy may be attributed to the fact that this study included only 10 healthy monks aged 25–65 years with a BMI > 30 kg/m^2^. Notably, the London Ramadan Study (LORANS), an observational, systematic review and meta-analysis study, supported evidence that Ramadan fasting can exert beneficial effects on blood pressure independently of changes in body weight, total body water and fat mass [49]. In this aspect, it should be noted that the decreased sedentary behavior of fasters may be associated with more time outdoors, including daytime under natural sun, which could be related to changes in chronotype and cardiometabolic health. Remarkably, a substantial systematic review reported that the chronotypes are highly related to physical activity levels and sedentary behavior, especially in the population over their mid-twenties [50]. Evening chronotypes seem to be related to lower physical activity and more time in sedentary activities compared to morning chronotypes [50,51].

One of the most significant findings of our study was the fact that fasting patients showed a significantly and independently lower prevalence of overweight/obesity and abdominal obesity than non-fasters based on three different anthropometric indices of high validity. In this aspect, there are currently several studies evaluating the potential effects of fasting on body mass and weight control in fasters and non-fasters. More to the point, a recent well-designed study conducted on 43 males (20–45 years old) from the general population who habitually fasted and 57 age-matched Athonian monks supported the evidence that monks exhibited a lower BMI, as well as a lower body fat mass [39]. Moreover, a 40-day (fasting and non-fasting days) longitudinal study including 36 nuns and monks revealed that fasting resulted in a reduction in the body weight, upper arm circumference and triceps skinfold thickness [41]. A reduction in monks’ body mass was also noted even at the first week of fasting, albeit not at a significant level [34]. Interestingly, a one-year follow-up study demonstrated that fasters showed a 1.5% lower BMI than non-fasters at the end of the fasting period, while a 1.4% decline in the BMI was also observed in fasters next to the fasting period [47]. A prospective study conducted on 140 individuals in Ethiopia also showed that Christian Orthodox can result in considerable decreases in body weight and compositions. A longer fasting duration resulted in much higher impacts on body weight and body composition, supporting evidence that it could be considered a non-pharmacological strategy for the prevention or treatment of chronic diseases, such as metabolic disorders [48]. Among our above results, the lower prevalence of abdominal obesity in fasters compared to in non-fasters deserves special attention, as it is well-established that abdominal obesity increases the risk of cardiovascular and metabolic diseases. In agreement with our results, a recent clinical study showed that Christian Orthodox fasting during Holy Week resulted in a decreased waist circumference and waist-to-hip ratio, as well as body weight, BMI, total body fat, blood glucose, total cholesterol and LDL cholesterol levels; however, most of these favorable changes returned to pre-fasting levels after fasting cessation [52]. Nevertheless, this study included a small sample size [52].

Hence, Christian Orthodox fasting seems to exert a beneficial effect on body weight control. In this respect, it should be noted that the present data concerning the high prevalence of abdominal obesity in non-fasters compared to fasters deserve special attention, as this type of obesity may strongly increase the risk of several cardiometabolic diseases, such as diabetes mellitus type 2, metabolic syndrome, myocardial infraction and stroke [53,54]. However, a recent cross-sectional study on 175 individuals aged >50 years showed no differences in anthropometric parameters among fasters and non-fasters, except for the hip circumference, with fasters having higher mean values than non-fasters [18]. Another study conducted on 134 postmenopausal women aged 57.3 ± 6.7 years demonstrated that postmenopausal female fasters had a significantly higher mean fat free mass, hip circumference and diastolic blood pressure, while no other differences were found with regards to their anthropometric data [55]. However, these discrepancies may be attributed to the fact that the study population was exclusively restricted to postmenopausal women [55].

Overall, we did not find significant nutritional deficiencies between fasters and non-fasters, even if some nutrients were lower in fasters, albeit not at a significant level. This nutritional adequacy was ensured by the inclusion of seafoods and snails in this traditional religious fasting. Most of the above findings are in agreement with previous studies, suggesting that Christian Orthodox fasting is a safe nutritional intervention to promote human health without leading to significant dietary deficiencies [9,10,56,57]. Although in some cases, fasters presented with a bit lower level of essential nutrients and lower serum levels of some blood biochemical parameters, they all remain between the physiological and recommended ranges [9,18,56,57]. The significant associations of a higher educational level and no smoking history, as well as lower sedentary behavior, in fasters compared to in non-fasters, are also in accordance with previous studies [9,18]. Accordingly, a recent cross-sectional study on 400 individuals aged 22.6 ± 17.0 years that followed Christian Orthodox fasting for at least 12 years showed that fasters had a significantly lower daily intake of calories and cholesterol compared with non-fasters. Furthermore, fasters reported a healthier way of living, with lower rates of smoking and alcohol consumption [58].

However, the present study has some limitations and disadvantages. The sample size is not sufficiently adequate to generalize the findings to either the general Greek population with the same religion and traditional dietary pattern or to other Christians populations of other ethnicities. In addition, due to the cross-sectional design of our study, no definitive conclusions about causality can be established. Moreover, due to the methodology, recall bias should be taken into consideration since sociodemographic and lifestyle factors were self-reported by participants. However, self-reported data have widely been used in epidemiological studies, presenting with adequate reliability and accuracy to estimate diverse outcomes. Moreover, the likelihood of unmeasured confounding factors, like several aspects of mental health and the presence of eating disorders of the assigned patients remains, despite of our systematic approaches to confounder adjustments. Thus, it remains quite possible that residual confounding factors may influence our results even if we have applied a thorough adjustment for multiple confounders. In addition, even if we collected all study data during a non-fasting period, we did not take the relevant data during a similar time of year in the exact season and an exact non-fasting phase according to the Christian Orthodox fasting timetable. Lastly, there is a significant demand to explore the potential positive effects of Christian Orthodox fasting on each metabolic disorder separately, as each of them has different pathophysiological characteristics, which may differentially affect the results of the present study.

The present study also has certain strengths. Firstly, the use of face-to-face interviews and the detailed description of the questionnaires to the participants can minimize the possibility of false responses, increasing the validity of the study. In addition, the use of measured and not self-reported anthropometric parameters enhances the validity of the present study. Notably, indices beyond the BMI, WC and WtHR were measured and subjected to analysis, and these are effective indices of abdominal obesity, which are directly associated with a higher risk of diverse chronic human diseases, including metabolic disorders. It should be noted that both WC and WtHR indices can provide a better representation of body fat distribution. Moreover, the fasting participants had adapted Christian Orthodox fasting recommendations based on the fasting calendar for a long and simultaneously adequate period, either from their childhood or at least for the last twelve consecutive years, which further reinforces our findings and increases their validity. Lastly, this is one of the few clinical studies examining the potential beneficial effects of this type of traditional religious fasting in association with several sociodemographic, anthropometric and lifestyle factors, in conjunction with a plethora of blood biochemical parameters that reflect the health status of the study population.

## 5. Conclusions

This is one of the few cross-sectional studies demonstrating that Christian Orthodox fasting, a traditional time-restricted religious fasting with a history of 2000 years and distinct difference from other related dietary patterns, may promote metabolic health by improving several aspects of the symptom severity of metabolic disorders. However, future well-designed prospective studies, including a larger study population from different countries and diverse ethnicities that follow Christian Orthodox fasting, are recommended to evaluate the impact of long-term religious fasting effects on human metabolic health, either as a preventing factor for reducing the risk of chronic diseases and especially cardiometabolic disorders or as a nutritional intervention to ameliorate the symptom severity of chronic and cardiometabolic diseases. Another strong demand is to investigate the potential beneficial effect of Christian Orthodox fasting on each metabolic disorder separately since each of them has specific pathological characteristics beyond the general background of metabolic disturbances.

## Figures and Tables

**Table 1:** Descriptive statistics of the enrolled study population during a non-fasting period.

Characteristics (n = 135)	Descriptive Statistics
**Age (mean ± SD; years)**	55.8 ± 7.3
**Gender (n, %)**	
Male	40 (29.6%)
Female	95 (70.4%)
**Educational level (n, %)**	
Primary education	32 (23.7%)
Secondary education	55 (40.7%)
University studies	88 (35.6%)
**Smoking (n, %)**	
Regular smokers	41 (30.4%)
Non-smokers	94 (69.6%)
**Marital status (n, %)**	
Married	105 (77.8%)
Not married	30 (22.2%)
**Physical activity (n, %)**	
Very low	56 (41.5%)
Low	37 (27.4%)
Moderate	29 (21.5%)
High	13 (9.6%)
**Sedentary behavior (n, %)**	
Low	61 (45.2%)
Moderate	47 (34.8%)
High	27 (20.0%)
**BMI (n, %)**	
Normal weight	18 (13.3%)
Overweight	64 (47.4%)
Obese	53 (39.3%)
**Abdominal obesity status (WC) (n, %)**	
Low	26 (19.2%)
Medium	46 (34.1%)
High	63 (46.7%)
**Abdominal obesity status (WtHR) (n, %)**	
Low	23 (17.0%)
Medium	58 (43.0%)
High	54 (40.0%)
**Sleep duration (n, %)**	
Normal (6–8 h)	71 (52.6%)
Abnormal (6 h ˂ or ˃ 8 h)	64 (47.4%)
**Disease (n, %)**	
Hypothyroidism	39 (28.9%)
Excessive obesity	31 (23.0%)
Osteoporosis	31 (23.0%)
Diabetes mellitus type 2	34 (25.1%)
**Fasting (n, %)**	
Yes	67 (49.6%)
No	68 (50.4%)
**Hypertension (n, %)**	
No	56 (41.5%)
Yes	79 (58.5%)
**Systolic pressure (** **mean ± SD;** **mm Hg)**	134.6 ± 11.4
**Diastolic pressure (** **mean ± SD;** **mm Hg)**	83.2 ± 5.8
**Albumin (** **mean ± SD; g/dL)**	4.2 ± 0.3
**Cholesterol (** **mean ± SD;** **mg/dL)**	205.8 ± 98.7
**Triglycerides (** **mean ± SD;** **mg/dL)**	179.9 ± 98.7
**LDL (** **mean ± SD;** **mg/dL)**	115.5 ± 43.8
**HDL (** **mean ± SD;** **mg/dL)**	50.7 ± 16.2
**Vitamin D 25-OH (** **mean ± SD;** **ng/mL)**	17.3 ± 7.4
**Folic acid (** **mean ± SD;** **ng/mL)**	2.04 ± 1.71
**Vitamin B12 (** **mean ± SD;** **pg/mL)**	315.2 ± 206.1
**CRP (** **mean ± SD;** **mg/dL)**	0.36 ± 0.29
**Glucose (** **mean ± SD;** **mg/dL)**	102.1 ± 35.6
**Ca (** **mean ± SD;** **mg/dL)**	9.6 ± 1.4
**ALP (** **mean ± SD;** **U/L)**	66.2 ± 18.8
**Fe (** **mean ± SD;** **μ** **g/dL)**	1.1 ± 28.7
**Creatinine (** **mean ± SD;** **mg/dL)**	0.9 ± 0.2
**Urea (** **mean ± SD; mg/dL)**	31.9 ± 14.1
**Uric acid (** **mean ± SD; mg/dL)**	4.8 ± 1.3

**Table 2:** Association of Christian Orthodox fasting with sociodemographic, anthropometric and lifestyle factors and biochemical indices.

Parameters (n = 135)	Christian Orthodox Fasting		*p*-Value
	Yes 67 (49.6%)	No 68 (50.4%)	
**Age (mean ± SD years)**	55.4 ± 7.5	55.1 ± 7.3	*p* = 0.5521
**Gender (n, %)**			*p* = 0.0464
Male	16 (23.9%)	24 (35.3%)	
Female	51(76.1%)	44 (64.7%)	
**Educational level (n, %)**			*p* = 0.0001
Primary education	10 (14.9%)	22 (32.3%)	
Secondary education	20 (29.9%)	35 (51.5%)	
University studies	37 (55.2%)	11 (16.2%)	
**Smoking (n, %)**			*p* = 0.0018
Regular smokers	12 (17.9%)	29 (42.7%)	
Non-smokers	55 (82.1%)	39 (57.3%)	
**Marital status (n, %)**			*p* = 0.9633
Married	52 (77.6%)	53 (77.9%)	
Not married	15 (22.4%)	15 (22.1%)	
**Physical activity (n, %)**			*p* = 0.0192
Very low	25 (37.3%)	31 (45.6%)	
Low	14 (20.9%)	23 (33.8%)	
Moderate	17 (25.4%)	12 (17.7%)	
High	11 (16.4%)	2 (2.9%)	
**Sedentary behavior (n, %)**			*p* = 0.0003
Low	17 (25.4%)	44 (64.7%)	
Moderate	32 (47.7%)	15 (22.1%)	
High	18 (26.9%)	9 (13.2%)	
**BMI (n, %)**			*p* = 0.0075
Normal weight	15 (22.4%)	3 (4.4%)	
Overweight	30 (44.8%)	34 (50.0%)	
Obese	22 (32.8%)	31 (45.6%)	
**Abdominal obesity status (WC) (n, %)**			*p* = 0.0017
Low	21 (31.3%)	5 (7.3%)	
Medium	18 (26.9%)	28 (41.2%)	
High	28 (41.8%)	35 (51.5%)	
**Abdominal obesity status (WtHR) (n, %)**			*p* ˂ 0.0001
Low	16 (23.9%)	7 (10.3%)	
Medium	41 (61.2%)	17 (25.0%)	
High	10 (14.9%)	44 (64.7%)	
**Sleep duration (n, %)**			*p* = 0.0437
Normal (7–8 h)	40 (59.7%)	31 (45.6%)	
Abnormal (7 h ˂ or ˃ 8 h)	27 (40.3%)	37 (54.4%)	
**Disease (n, %)**			*p* = 0.2532
Hypothyroidism	23 (34.3%)	16 (23.5%)	
High obesity	16 (23.9%)	15 (22.1%)	
Osteoporosis	16 (23.9%)	15 (22.1%)	
Diabetes mellitus type 2	12 (17.9%)	22 (32.3%)	
**Hypertension (n, %)**			*p* = 0.0004
No	38 (56.7%)	18 (26.5%)	
Yes	29 (43.3%)	50 (73.5%)	
**Systolic pressure (mean ± SD; mmHg)**	130.9 ± 9.7	138.2 ± 11.9	*p* = 0.0001
**Diastolic pressure (mean ± SD; mmHg)**	81.2 ± 5.3	85.1 ± 5.7	*p* ˂ 0.0001
**Albumin (mean ± SD; g/dL)**	4.3 ± 0.2	4.2 ± 0.3	*p* = 0.0118
**Cholesterol (mean ± SD; mg/dL)**	203.8 ± 46.8	207.6 ± 38.1	*p* = 0.6074
**Triglycerides (mean ± SD; mg/dL)**	176.3 ± 106.7	183.5 ± 90.8	*p* = 0.6753
**LDL (mean ± SD; mg/dL)**	111.2 ± 45.2	119.8 ± 42.4	*p* = 0.2608
**HDL (mean ± SD; mg/dL)**	54.5 ± 16.9	47.0 ± 14.7	*p* = 0.0068
**Vitamin D 25-OH (mean ± SD; ng/mL)**	17.2 ± 8.0	17.4 ± 6.8	*p* = 0.7448
**Folic acid (mean ± SD; ng/mL)**	1.9 ± 1.5	2.1 ± 1.9	*p* = 0.4243
**Vitamin B12 (mean ± SD; pg/mL)**	312.2 ± 210.6	315.3 ± 203.2	*p* = 0.7967
**CRP (mean ± SD; mg/dL)**	0.31 ± 0.29	0.41 ± 0.30	*p* = 0.0367
**Glucose (mean ± SD; mg/dL)**	95.3 ± 25.6	109.0 ± 42.4	*p* = 0.0202
**Ca (mean ± SD; mg/dL)**	9.4 ± 1.9	9.7 ± 0.6	*p* = 0.1763
**ALP (mean ± SD; U/L)**	68.7 ± 17.6	63.7 ± 19.7	*p* = 0.1213
**Fe (mean ± SD;** **μ** **g/dL)**	88.3 ± 27.6	93.9 ± 29.6	*p* = 0.2614
**Creatinine (mean ± SD; mg/dL)**	0.88 ± 0.19	0.92 ± 0.14	*p* = 0.2081
**Urea (mean ± SD; mg/dL)**	31.1 ± 11.6	32.7 ± 16.3	*p* = 0.4889
**Uric acid (mean ± SD; mg/dL)**	4.7 ± 1.3	5.1 ± 1.4	*p* = 0.1162

**Table 3:** Multivariate binary logistic regression analysis of Christian Orthodox fasting adjusting for multiple confounding factors.

Characteristics	Christian Orthodox Fasting (Yes vs. No)	
	HR * (95% CI **)	*p*-Value
**Age** (Over/Below mean value)	1.18 (0.72–1.73)	*p* = 0.1256
**Gender** (Male/Female)	1.23 (0.68–1.85)	*p* = 0.3601
**Educational level** (University studies/Primary or secondary education)	1.81 (1.47–2.08)	*p* = 0.0024
**Smoking** (Non-smokers/Regular smokers)	1.72 (1.38–2.11)	*p* = 0.0103
**Marital status** (Married/Not married)	0.96 (0.25–1.68)	*p* = 0.9458
**Physical activity** (Moderate and high/Very low and low)	1.19 (0.71–1.96)	*p* = 0.3946
**Sedentary behavior** (Low/Moderate and high)	1.31 (1.03–1.52)	*p* = 0.0185
**BMI** (Normal weight/Overweight and obese)	1.82 (1.50–2.19)	*p* = 0.0293
**WC** (Low and medium/high)	1.95 (1.71–2.18)	*p* = 0.0128
**WtHR** (Low and medium/high)	2.05 (1.86–2.27)	*p* = 0.0002
**Sleep duration** (Normal/Abnormal)	1.05 (0.58–1.63)	*p* = 0.1879
**Hypertension** (No/Yes)	1.67 (1.39–1.95)	*p* = 0.0088
**Systolic pressure** (Below vs. above mean value)	1.58 (1.30–1.85)	*p* = 0.0007
**Diastolic pressure** (Below vs. above mean value)	1.72 (1.47–1.98)	*p* = 0.0005
**Albumin** (Below vs. above mean value)	1.32 (0.87–1.79)	*p* = 0.1032
**Cholesterol** (Below vs. above mean value)	1.05 (0.48–1.57)	*p* = 0.7253
**Triglycerides** (Below vs. above mean value)	1.11 (0.65–1.68)	*p* = 0.6976
**LDL** (Below vs. above mean value)	1.08 (0.59–1.61)	*p* = 0.4832
**HDL** (Below vs. above mean values)	0.85 (0.49–1.18)	*p* = 0.0231
**Vitamin D 25-OH** (Below vs. above mean values)	0.96 (0.51–1.52)	*p* = 0.5428
**Folic acid** (Below vs. above mean values)	0.92 (0.59–1.57)	*p* = 0.5011
**Vitamin B12** (Below vs. above mean values)	0.95 (0.54–1.47)	*p* = 0.6129
**CRP** (Below vs. above mean values)	0.88 (0.51–1.22)	*p* = 0.0866
**Glucose** (Below vs. above mean values)	0.86 (0.53–1.19)	*p* = 0.0402
**Ca** (Below vs. above mean values)	0.92 (0.49–1.52)	*p* = 0.3743
**ALP** (Below vs. above mean values)	1.02 (0.47–1.53)	*p* = 0.5457
**Fe** (Below vs. above mean values)	1.13 (0.67–1.78)	*p* = 0.4828
**Creatinine** (Below vs. above mean values)	1.04 (0.55–1.63)	*p* = 0.5732
**Urea** (Below vs. above mean values)	1.06 (0.49–1.53)	*p* = 0.5821
**Uric acid** (Below vs. above mean values)	0.95 (0.57–1.51)	*p* = 0.3023

* Hazard Ratio: HR; ** CI: Confidence Interval.

## Data Availability

The data of the present study are available upon request to the corresponding author due to private policy.
